# Investigating the Anti-Inflammatory Properties and Skin Penetration Ability of Cornelian Cherry (*Cornus mas* L.) Extracts

**DOI:** 10.3390/ijms25094763

**Published:** 2024-04-27

**Authors:** Martyna Zagórska-Dziok, Anna Nowak, Anna Muzykiewicz-Szymańska, Aleksandra Ziemlewska, Zofia Nizioł-Łukaszewska, Agnieszka Mokrzyńska, Magdalena Wójciak, Ireneusz Sowa

**Affiliations:** 1 Department of Technology of Cosmetic and Pharmaceutical Products, Medical College, University of Information Technology and Management in Rzeszow, Sucharskiego 2, 35-225 Rzeszow, Poland; mazagorska@wsiz.edu.pl (M.Z.-D.); aziemlewska@wsiz.edu.pl (A.Z.); zniziol@wsiz.edu.pl (Z.N.-Ł.); amokrzynska@wsiz.edu.pl (A.M.); 2 Department of Cosmetic and Pharmaceutical Chemistry, Pomeranian Medical University in Szczecin, 70-111 Szczecin, Poland; anowak@pum.edu.pl (A.N.); anna.muzykiewicz@pum.edu.pl (A.M.-S.); 3 Department of Analytical Chemistry, Medical University of Lublin, Aleje Raclawickie 1, 20-059 Lublin, Poland; magdalena.wojciak@umlub.pl

**Keywords:** antioxidant activity, *Cornus mas* L., dogwood, fibroblasts, keratinocytes, cytotoxicity, pro-inflammatory cytokines, skin permeation

## Abstract

Plant extracts can be a valuable source of biologically active compounds in many cosmetic preparations. Their effect depends on the phytochemicals they contain and their ability to penetrate the skin. Therefore, in this study, the possibility of skin penetration by phenolic acids contained in dogwood extracts of different fruit colors (yellow, red, and dark ruby red) prepared using different extractants was investigated. These analyses were performed using a Franz chamber and HPLC-UV chromatography. Moreover, the antioxidant properties of the tested extracts were compared and their impact on the intracellular level of free radicals in skin cells was assessed. The cytotoxicity of these extracts towards keratinocytes and fibroblasts was also analyzed and their anti-inflammatory properties were assessed using the enzyme-linked immunosorbent assay (ELISA). The analyses showed differences in the penetration of individual phenolic acids into the skin and different biological activities of the tested extracts. None of the extracts had cytotoxic effects on skin cells in vitro, and the strongest antioxidant and anti-inflammatory properties were found in dogwood extracts with dark ruby red fruits.

## 1. Introduction

*Cornus mas* L. (*C. mas* L.) is a very popular plant that has been used for centuries to treat various types of diseases [1]. However, there are no reports in the literature on the possibility of penetration of the active substances contained in these fruits through the skin. Our previous research confirmed its antioxidant, antibacterial, anti-inflammatory, and anti-aging effects [2,3,4]. These properties therefore indicate that *C. mas* can be an extremely valuable raw material used in the development of new recipes for cosmetic preparations. Due to the existence of many different varieties of this plant, this study attempted to compare the biological activity of three dogwood varieties in order to select the variety that could be the raw material with the most beneficial effect on the skin. Additionally, this is the first work that assesses the possibility of penetration or accumulation of *C. mas* phytochemicals in the skin. This knowledge can help assess in which layers of the skin individual compounds may exhibit biological activity. Assessment of the penetration of active substances through the skin or their accumulation in it is very important, especially when designing new cosmetic or dermatological preparations containing plant raw materials. The ability of biologically active substances present in dogwood fruit to penetrate deeper layers of the skin may contribute to their action in all layers of the skin. On the other hand, the possibility of accumulation of selected phytochemicals in individual layers may contribute to the extension of their action. The possibility of penetration is closely related to the chemical nature of the compounds present in plant extracts, primarily their hydrophilicity and lipophilicity [5,6]. Plant extracts contain a number of secondary metabolites with antioxidant activity that may have a beneficial effect on the skin and its deeper layers [7]. Natural plant antioxidants can effectively control oxidative stress through their multidirectional action [8]. Reactive oxygen species (ROS) act primarily by several important molecular pathways that play essential protection roles in skin inflammation or development of infection [9]. Moreover, in the case of dermocosmetics containing plant extracts, the solvent used can significantly affect the release and penetration of active substances through the skin [10]. Differences in the content of basic secondary metabolites in fruits may vary depending on the variety of the fruit [11].

Various types of products based on *C. mas* L. extracts have been developed so far, both by the pharmaceutical and food industries. These are mainly based on fruits, but leaves, flowers, seeds, and bark are also used to prepare products from this plant. Dogwood products are used to eliminate stomach problems and support the proper function of the liver, pancreas, and kidneys. The fruits of this plant also support the treatment of various viral diseases, such as measles or chickenpox, and help achieve better results in the treatment of rickets and diabetes [1,12]. Dogwood berries are also a valuable raw material in the cosmetics industry due to their toning and astringent properties. Moreover, these fruits may have anti-inflammatory and anti-aging properties and inhibit the proliferation of various cancer cells [13]. This raw material is stored mainly in the form of dried or freeze-dried preparations, which provides greater resistance to microorganisms and enzymatic changes [13,14]. 

A wide spectrum of compounds present in the fruits of *C. mas* L. undoubtedly play an extremely important role in the multidirectional activity of extracts from these fruits. Due to the fact that many skin diseases are associated with the co-occurrence of inflammation, when assessing the possibility of using these extracts in preparations intended for the care and elimination of skin imperfections, the assessment of pro- or anti-inflammatory properties is extremely important [15]. Differences in both the antioxidant and anti-inflammatory activity of dogwood varieties with different fruit colors are related to the different content of biologically active compounds such as polyphenols, anthocyanins, flavonoids, and iridoids [16]. Phenolic compounds are extremely valuable bioactive compounds of plant origin with a wide range of biological properties [2]. They have a positive effect on health mainly due to their antioxidant properties and the ability to inhibit inflammatory processes [17]. The antioxidant properties of this group of compounds are related to their molecular structure; hence, different phenolic acids may have different properties [18]. Polyphenols have the ability to impair the nuclear factor κB (NF-κB) pathway, which plays a very important role in the development of the inflammatory response. This is due to the promotion of the expression of adhesion molecules, cytokines, and other pro-inflammatory mediators [19,20]. The anti-inflammatory effects of these phytochemicals may also include inhibition of C-reactive protein (CRP) and pro-inflammatory mediators such as interleukin 6 (IL-6) and tumor necrosis factor alpha (TNF-α) [21,22]. Other authors also pointed out the possibility of polyphenols modulating the synthesis of inflammatory cytokines, such as interleukin 1β (IL-1β), interleukin 2 (IL-2), and interleukin 6 (IL-6) [23].

Polyphenols are phytochemicals that can influence the proliferation and metabolic activity of various types of human cells and control the proliferation of bacterial cells [24,25,26]. Therefore, when including plant extracts rich in polyphenol compounds in the formulation of preparations applied directly to the skin, it is very important to assess their cytotoxicity towards skin cells. Available data from the literature indicate that many phenolic compounds have a positive effect on skin cells and can be used in the treatment of a wide range of skin diseases. This effect was confirmed by both in vitro and in vivo studies, indicating the different mechanisms of action of this group of phytochemicals [27,28]. Scientific research also demonstrates a positive effect of biologically active compounds present in dogwood fruits on skin cells, which indicates their potential use in cosmetology and dermatology [2,29]. The difference in the content of biologically active compounds in dogwood fruits of different colors may significantly affect the biological activity of the obtained extracts. Selecting the appropriate variety of plant raw material allows to obtain a product with the desired properties. Due to differences in the solubility of individual phytochemicals in various solvents, selecting the appropriate extraction medium allows for the extraction of specific chemical compounds that will be responsible for the biological activity of individual cosmetic preparations. The aim of this study was to compare the biological activity of extracts obtained from three varieties of *C. mas* L. and the possibility of their penetration through the skin. For this purpose, the biological properties of various types of extracts prepared from dogwood varieties with yellow, red, and dark red fruits were assessed. The antioxidant and anti-inflammatory properties of the tested extracts were analyzed and their impact on the viability of keratinocytes and fibroblasts in vitro was determined. The research was also aimed at identifying dogwood extracts with the most desirable properties in the context of their use in dermatology and cosmetology.

## 2. Results and Discussion

### 2.1. HPLC Analysis

Very important ingredients of natural cosmetic preparations are phenolic acids belonging to the group of polyphenols. These substances are valuable metabolites due to their, among others, antioxidant, antibacterial, anticarcinogenic, and anti-inflammatory properties [30,31]. 

The content of individual phenolic acids in all analyzed extracts from *C. mas* L. is presented in Table 1, Table 2 and Table 3. The following phenolic acids were identified in the tested extracts: gallic acid, protocatechuic acid, gentisic acid, *p*-hydroxybenzoic acid, hypogallic acid, *m*-salicylic acid, caffeic acid, and chlorogenic acid. As a result of the research, statistically significant differences were observed in the amount of phenolic compounds between individual extracts. All analyzed extracts had the highest content of gallic acid. In the case of the dark ruby red-fruited dogwood variety (DRV), the highest content of gallic acid was observed in DRE30; in the red-fruited variety (RV), it was in RE70; while in the yellow-fruited variety (YV), it was in YE30. Similarly, the highest amounts of gallic acid in dogwood fruit were identified in another study that analyzed 23 Cornelian cherry genotypes growing in the wild in the Eastern Anatolia Region [32]. Close values of this compound were identified in a previous study in which water, water-glyceric and water-ethanolic extracts were analyzed. The tested extracts contained gallic acid in amounts ranging from 9.73 ± 0.41 to 36.41 ± 1.91 µg/mL [2]. While, in another study, characterization of 11 Montenegrin local or introduced genotypes and cultivars collected in the wild or from organic orchards is provided, wherein gallic acid was significantly highest in the local genotypes (6.80 ± 0.36 mg/100 g) [33]. Our study also identified a high content of protocatechic and chlorogenic acids. YE30 contained the highest amount of both acids, 35.45 ± 2.18 and 33.01 ± 1.35 µg/mL, respectively. Both protocatechuic acid and chlorogenic acid have often been previously identified in dogwood fruit. Both protocatechuic acid and chlorogenic acid in dogwood fruit have often been previously identified in other studies [18,32,33]. The content of phenolic acids in the plant extract is undoubtedly influenced by the type and concentration of the solvent used, whereas solvents with low viscosity are more effective because they easily penetrate plant material. In our study, extracts prepared in ethanol–water solvents, in both concentrations used, had the highest effectiveness. In most cases, water extracts showed lower contents of the analyzed compounds. Similarly, the highest amount of phenolic compounds in *C. mas* L. fruit extract was observed in an extract obtained from a solvent of water–ethanol (50:50 *v*/*v*) [34]. Ethanol and its mixtures with water are good solvents and are often chosen for the extraction of phenolic acids from plant material [35]. 

### 2.2. Antioxidant Capacity Using DPPH and Folin–Ciocalteu Methods

Due to their high antioxidant activity, phenolic acids are considered to be crucial in skin protection against oxidative stress [36]. The skin is constantly exposed to oxidative stress induced by reactive oxygen species (ROS), damaging cellular constituents, such as cell membrane lipids, proteins, or DNA [10]. Therefore, it is very important to support the skin endogenous protective system, and this can be achieved by using the exogenous antioxidants included in fruits [37]. 

**Figure 1:**
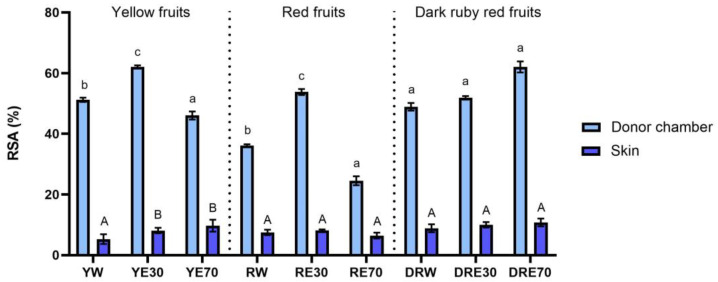
The antioxidant activity of the extract applied to the skin and solution obtained after skin extraction after 24 h of penetration (*n* = 3). Different letters indicate significant differences between individual extracts within each *C. mas* L. variety (small letters—extracts applied to the skin, big letters—solutions obtained after skin extraction), α = 0.05. The acceptor fluid collected after 24 h of penetration did not show any antioxidant activity.

**Figure 2:**
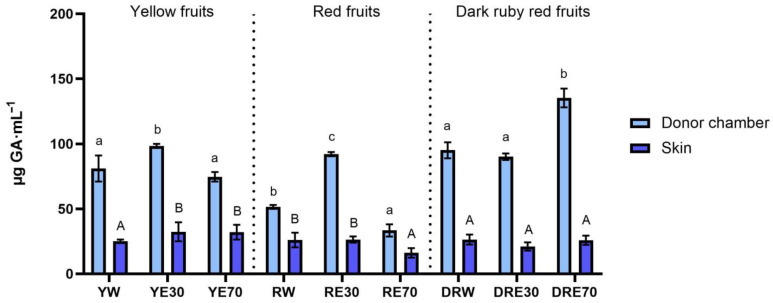
The total polyphenol content of the extract applied to the skin and solution obtained after skin extraction after 24 h of penetration (*n* = 3). Different letters indicate significant differences between individual extracts within each *C. mas* L. variety. (small letters—extracts applied to the skin, big letters—solutions obtained after skin extraction), α = 0.05. The acceptor fluid collected after 24 h of penetration did not show any total polyphenol content.

**Figure 3:**
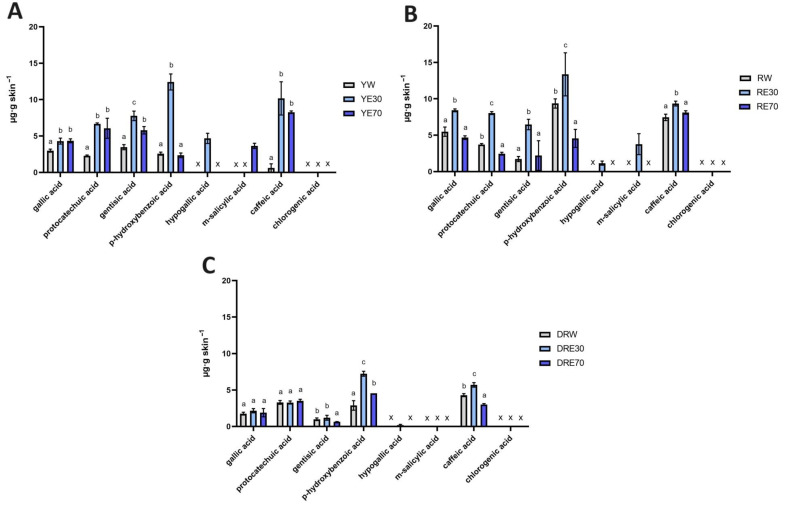
The content of phenolic acids in the skin extracts obtained after 24 h penetration study in *C. mas* L. The figure shows the results for the extracts from the cultivar with yellow fruit (**A**), with red fruit (**B**) and with dark ruby red fruit (**C**); x—no identified phenolic acids. Analyses were carried out for yellow fruit extracts (water (YW), water–ethanol 30:70 (YE30), water–ethanol 70:30 (YE70)), red fruit extracts (water (RW), water–ethanol 30:70 (RE30), water–ethanol 70:30 (RE70)) and dark ruby red extracts (water (DRW), water–ethanol 30:70 (DRE30), water–ethanol 70:30 (DRE70)). Different letters indicate significant differences between individual extracts within each *C. mas* L. variety; (*n* = 3); α = 0.05.

In our study, we demonstrated the antioxidant activity in two compartments: (1) the plant extract applied to the skin, and (2) the fluid obtained after skin extraction, collected after the completion of penetration. The antioxidant activity and total content of polyphenols in extracts applied to the skin and extracts after extraction from the skin collected after a 24 h examination are shown in Figure 1. All extracts applied to the skin showed the ability to scavenge free radicals, measured by the DPPH method. In the case of the DRV, DRE70 were characterized by significantly higher antioxidant activity (62.05 ± 1.80% RSA); however, in the case of the remaining varieties, the highest significant antioxidant capacity was demonstrated by RE30 (53.79 ± 1.48% RSA) and YE30 (62.05 ± 0.49% RSA). However, the highest total content of polyphenols was contained in the DRE70 extract (Figure 2). Whereas, in the extracts applied to the skin, the content of the polyphenols ranges from 33.37 ± 4.70 µg·mL^−1^ for the RE70 to 135.25 ± 7.22 µg·mL^−1^ for the DRE70 (Figure 3). Many previous studies report high antioxidant activity of *C. mas* L. fruits extracts [2,13,29,38]; therefore, this fruit can be used as an ingredient in preparations providing antioxidants to the skin. In the case of some plant substances, contained in cosmetics preparations, their greater accumulation in the skin is preferred, as, through their antioxidant activity, they could show, among others, anti-aging and anti-inflammatory effects [39]. In our study, the fluid obtained after extraction from the skin, dismantled after 24 h of penetration, also showed antioxidant activity. The percentage ability of extracts to scavenge free radicals when contained in the skin ranged from 5.31 ± 1.59 to 10.80 ± 1.29% RSA for YW and DRE70, respectively. The antioxidant activity of the fluid obtained after skin extraction indicated the accumulation of ingredients responsible for the antioxidant effect. Nowak et al. and Alonso et al. also demonstrated the high antioxidant activity of the skin’s extract evaluated by the DPPH test after applying compounds with a high antioxidant potential such as rutin, quercetin, or *E. angustifolium* extracts [10,40]. 

### 2.3. Permeation Skin Studies

In our study, a high accumulation of individual phenolic acids in the skin was also observed. Phenolic acid’s accumulations are shown in Figure 3. Phenolic acids accumulated to varying degrees depending on the *C. mas* L. variety and the solvent used. Not all analyzed compounds accumulated in the skin, but when comparing individual extracts, we can observe similar trends. For example, gallic acid, protocatechuic acid, gentisic acid, *p*-hydroxybenzoic acid and *m*-salicylic acid accumulated in the skin after the application of all analyzed extracts. However, hypogallic acid only accumulated after using water–EtOH extracts (30:70), while chlorogenic acid did not accumulate. In the case of the DRV, *p*-hydroxybenzoic acid and caffeic acid from the DRE30 accumulated in the highest amounts, which differed significantly from other extracts of this variety. In the red-fruited *C. mas* L. variety, the highest accumulation was also observed after application of RE30 to the skin. In the case of this extract, all compounds accumulated in the skin were in significantly higher amounts compared to the other solvents used, with the highest amounts accumulating as follows: *p*-hydroxybenzoic acid, caffeic acid, gallic acid, and protocatechuic acid. Similarly, in the case of the YV, the highest significant extract accumulations were also observed after the use of YE30 and were *p*-hydroxybenzoic acid, caffeic acid, and gentisic acid (Figure 3).

The herbal extracts contain a lot of valuable antioxidants, which can accumulate in the skin or penetrate deeper layers and systemic circulation. In the case of cosmetic preparations applied to the skin, it is important that valuable secondary metabolites accumulate in the skin for as long as possible, demonstrating a caring effect [39]. Therefore, testing the penetration into and through the skin is a very important aspect in assessing the effectiveness of natural cosmetics. The cumulative mass in the acceptor fluid, after 24 h penetration, is presented in Table 4, Table 5 and Table 6. In our studies, only some phenolic acids penetrated through the skin. The acids that penetrated from all the extracts analyzed were gallic acid and protocatechuic acid, whereas gentisic acid penetrated from all extracts of the DRV and the YV. While hydroxybenzoic acid, in the case of the DRV, only penetrated from water extract and in the case of YV, only from the YE70 (Table 4, Table 5 and Table 6). The type of solvent used to prepare the extractants probably had an influence on the penetration of phenolic acids into the skin. It was observed that, in most varieties, phenolic acids penetrated from water–ethanol extracts, in particular from water–ethanol (30:70). The slightly greater penetration after applying alcohol solutions to the skin is caused by the release of ethanol onto the stratum corneum of the epidermis and, as a consequence, by affecting the cells between the cellular cement. This results in loosening the lipid layer and, as a consequence, increases the diffusion of active compounds [10]. Therefore, ethanol alcohol is often used as a promoter of transepidermal transport, which affects the effectiveness of active substance penetration into the skin [5]. However, in our studies, not all phenolic acids penetrated the skin, and some were identified in the acceptor fluid only after 24 h of testing. The low penetration of some phenolic acids through the skin was confirmed by Bertges et al., who analyzed the release of secondary metabolites from a hydrogel containing 5% coffee seed extract [39]. In another study, rosmarinic acid did not penetrate human skin either from a water solution or from an ethanol/PG solution prepared from *Plectranthus ecklonii*. In this case, the permeation examination was carried out for 48 h. The authors explained such results, among other explanations, by the influence of other plant components, which could probably inhibit the penetration of the analyzed compound. These authors also indicate that cosmetic preparations should not cross the skin barrier, which is desirable for safety reasons [41].

### 2.4. Detection of Intracellular Levels of Reactive Oxygen Species (ROS)

The balance between the rate of free radical production and the concentration of antioxidants and the activity of protective enzymes neutralizing ROS determines the level of reactive oxygen species in the body and the speed of their reaction with cell components. Excessive accumulation of reactive oxygen species leads to structural and functional changes in the skin, including increased production of pro-inflammatory cytokines or disruption of the extracellular matrix [42,43]. 

Therefore, a detailed assessment of the antioxidant potential is very important. It has been shown that the antioxidative effect of the active substances contained in plant extracts may contribute to inhibiting or extinguishing free radical reactions [44,45,46]. Moreover, it should be remembered that the substances with antioxidant properties protect the lipids of the cell cement against oxidation. This happens at the initiation stage in the stratum corneum of the epidermis, where antioxidant compounds act mostly against free radicals. In the deeper parts of the epidermis, antioxidants can influence the activity of individual enzymes, and in the dermis, they can influence the condition of blood vessels and stimulate skin microcirculation [47]. The ability of dogwood to neutralize free radicals is closely related to the content of polyphenolic compounds, ascorbic acid, iridoids, and carotenoids [48,49]. Ersoy et al. showed that the ethanol extract from some varieties of dogwood fruit from Turkey has a significant H_2_O_2_-scavenging effect [50]. According to Seeram et al., cyanidin and pelargonidin galactosides present in dogwood extracts can inhibit lipid peroxidation [51]. Other authors also observed a protective effect on catalase and glutathione peroxidase by the polysaccharides contained in the fruit extracts of this plant [52]. 

**Figure 4:**
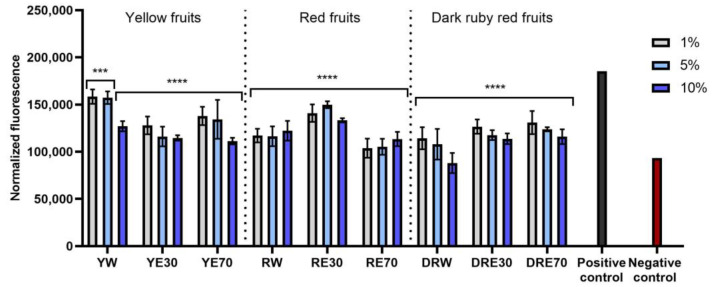
The effect of *C. mas* L. extracts with yellow, red, and dark ruby red fruits on the intracellular level of reactive oxygen species in fibroblasts (HDF) exposed to 500 μM H_2_O_2_. Positive controls are fibroblasts treated with H_2_O_2_ (without the addition of extracts) and negative controls are HDF cells treated neither with H_2_O_2_ nor extracts. Analyses were carried out for yellow fruit extracts (water (YW), water–ethanol 30:70 (YE30), water–ethanol 70:30 (YE70)), red fruit extracts (water (RW), water–ethanol 30:70 (RE30), water–ethanol 70:30 (RE70)) and dark ruby red extracts (water (DRW), water–ethanol 30:70 (DRE30), water–ethanol 70:30 (DRE70)). Data are the means ± SDs of three independent experiments in which each sample was tested in three replicates. **** *p* < 0.0001, *** *p* < 0.001.

**Figure 5:**
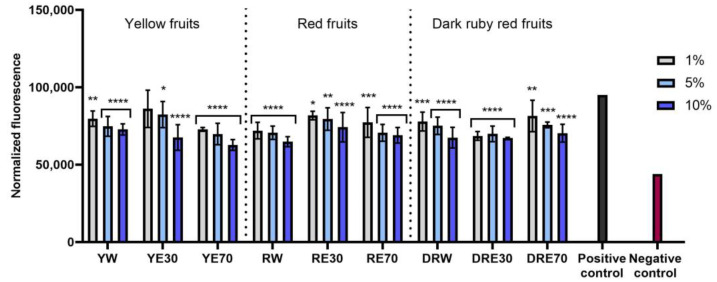
The effect of *C. mas* L. extracts with yellow, red, and ruby red fruits on the intracellular level of reactive oxygen species in keratinocytes (HaCaT) exposed to 500 μM H_2_O_2_. Positive controls are keratinocytes treated with H_2_O_2_ (without the addition of extracts) and negative controls are HaCaT cells treated neither with H_2_O_2_ nor extracts. Analyses were carried out for yellow fruit extracts (water (YW), water–ethanol 30:70 (YE30), water–ethanol 70:30 (YE70)), red fruit extracts (water (RW), water–ethanol 30:70 (RE30), water–ethanol 70:30 (RE70)) and dark ruby red extracts (water (DRW), water–ethanol 30:70 (DRE30), water–ethanol 70:30 (DRE70)). Data are the means ± SDs of three independent experiments in which each sample was tested in three replicates. **** *p* < 0.0001, *** *p* < 0.001, ** *p* = 0.0025, * *p* < 0.05.

The conducted research assessed the ability of dogwood extracts to generate intracellular production of reactive oxygen species in keratinocytes (HaCaT) and fibroblasts (HDF) cell lines. These analyses were performed using the fluorogenic dye H_2_DCFDA, which, in the presence of reactive oxygen species, is oxidized and converted into the highly fluorescent 2′,7′-dichlorofluorescein (DCF). Cells treated with 500 µM hydrogen peroxide (H_2_O_2_) were used as a positive control. The analyses performed showed that all types of extracts used in the experiment have the ability to reduce oxidative stress in both HDF and HaCaT cells. In almost all cases, the effect was dependent on the concentration used and increased with increasing concentration of the extracts. When HDF cells were exposed to RV extracts, increasing the concentration of the extracts did not result in such a clearly visible increase in the ability to reduce the intracellular level of free radicals (Figure 4). In the case of HDF cells, RE70 and DRW showed the highest potential for reducing intracellular oxidative stress. In the case of HaCaT cells, the strongest antioxidant activity was observed in the case of YE70, RW and DRE30. In the case of keratinocytes, a clear tendency to increase the ability to reduce the intracellular level of free radicals with increasing extract concentration was also observed (Figure 5).

### 2.5. Cytotoxicity Analysis

To evaluate the potential cytotoxicity of the examined extracts, the Alamar Blue and Neutral Red assays were employed. These assays facilitated the evaluation of the tested extracts that influenced the metabolic activity and proliferation of normal human skin cells—fibroblasts (HDF) and keratinocytes (HaCaT). The Alamar Blue (AB) assay involves the conversion of non-fluorescent resazurin to fluorescent resorufin, allowing assessment of cell viability, proliferation, and mitochondrial respiratory activity. On the other hand, the Neutral Red (NR) assay relies on the uptake of the neutral red dye by living cells into lysosomes, which is contingent upon the cells’ ability to maintain pH gradients, closely associated with adenosine triphosphate (ATP) production [53,54].

**Figure 6:**
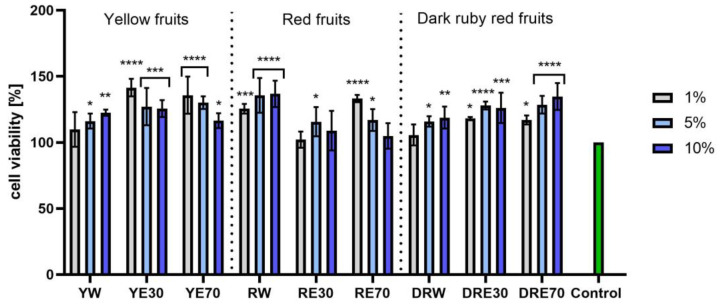
The effect of *C. mas* L. extracts with yellow, red, and dark ruby red fruits on the reduction in resazurin by fibroblasts (HDF). Controls are fibroblasts not treated with extracts, for which viability was assumed to be 100%. Analyses were carried out for yellow fruit extracts (water (YW), water–ethanol 30:70 (YE30), water–ethanol 70:30 (YE70)), red fruit extracts (water (RW), water–ethanol 30:70 (RE30), water–ethanol 70:30 (RE70)) and dark ruby red extracts (water (DRW), water–ethanol 30:70 (DRE30), water–ethanol 70:30 (DRE70)). Data are the means ± SDs of three independent experiments in which each sample was tested in three replicates. **** *p* < 0.0001, *** *p* < 0.001, ** *p* < 0.01, * *p* < 0.05.

**Figure 7:**
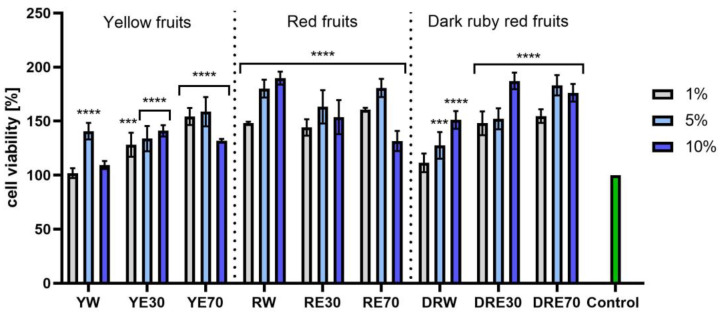
The effect of *C. mas* L. extracts with yellow, red, and dark ruby red fruits on the uptake of Neutral Red dye in fibroblasts (HDF). Controls are fibroblasts not treated with extracts, for which viability was assumed to be 100%. Analyses were carried out for yellow fruit extracts (water (YW), water–ethanol 30:70 (YE30), water–ethanol 70:30 (YE70)), red fruit extracts (water (RW), water–ethanol 30:70 (RE30), water–ethanol 70:30 (RE70)) and dark ruby red extracts (water (DRW), water–ethanol 30:70 (DRE30), water–ethanol 70:30 (DRE70)). Data are the means ± SDs of three independent experiments in which each sample was tested in three replicates. **** *p* < 0.0001, *** *p* < 0.001.

**Figure 8:**
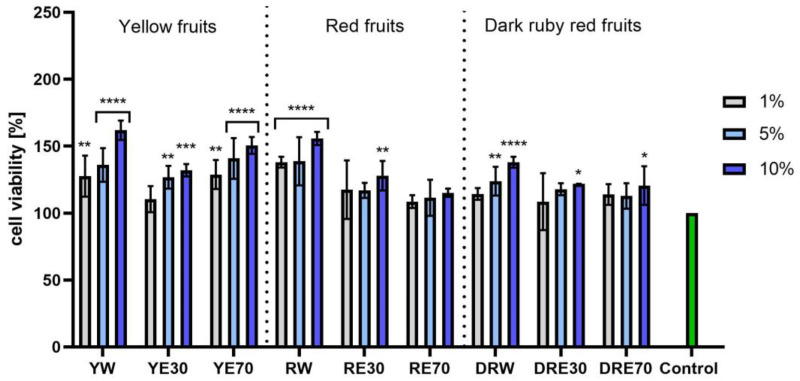
The effect of *C. mas* L. extracts with yellow, red, and dark ruby red fruits on the reduction in resazurin by keratinocytes (HaCaT). Controls are keratinocytes not treated with extracts, for which viability was assumed to be 100%. Analyses were carried out for yellow fruit extracts (water (YW), water–ethanol 30:70 (YE30), water–ethanol 70:30 (YE70)), red fruit extracts (water (RW), water–ethanol 30:70 (RE30), water–ethanol 70:30 (RE70)) and dark ruby red extracts (water (DRW), water–ethanol 30:70 (DRE30), water–ethanol 70:30 (DRE70)). Data are the means ± SDs of three independent experiments in which each sample was tested in three replicates. **** *p* < 0.0001, *** *p* = 0.0003, ** *p* < 0.01, * *p* < 0.05.

**Figure 9:**
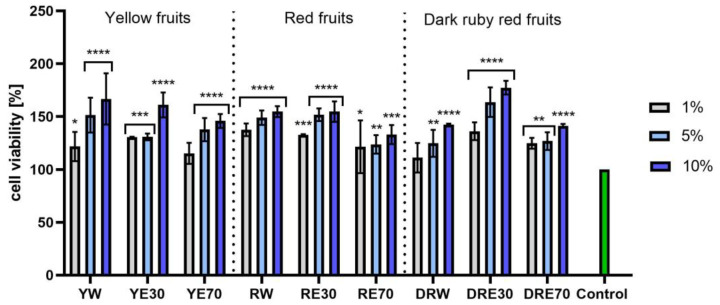
The effect of *C. mas* L. extracts with yellow, red, and dark ruby red fruits on the uptake of Neutral Red dye in keratinocytes (HaCaT). Controls are keratinocytes not treated with extracts, for which viability was assumed to be 100%. Analyses were carried out for yellow fruit extracts (water (YW), water–ethanol 30:70 (YE30), water–ethanol 70:30 (YE70)), red fruit extracts (water (RW), water–ethanol 30:70 (RE30), water–ethanol 70:30 (RE70)) and dark ruby red extracts (water (DRW), water–ethanol 30:70 (DRE30), water–ethanol 70:30 (DRE70)). Data are the means ± SDs of three independent experiments in which each sample was tested in three replicates. **** *p* < 0.0001, *** *p* < 0.001, ** *p* < 0.01, * *p* < 0.05.

**Figure 10:**
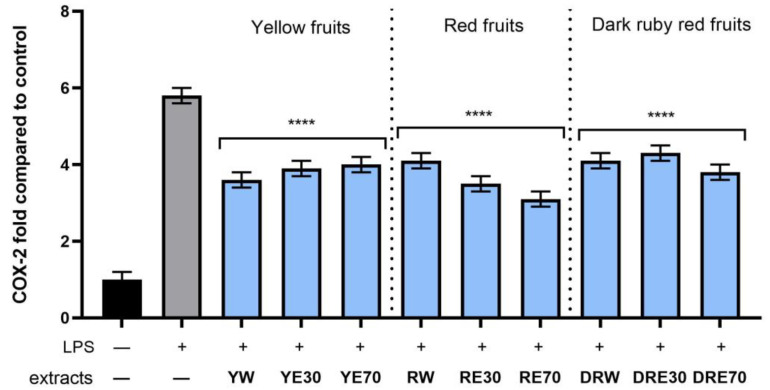
The effect of pretreatment with extracts of *C. mas* L. with yellow, red, and dark ruby red fruits before exposure to bacterial LPS (10 μg/mL) on the level of cyclooxygenase-2 (COX-2) calculated as a percentage in comparison with the untreated control. Analyses were carried out for yellow fruit extracts (water (YW), water–ethanol 30:70 (YE30), water–ethanol 70:30 (YE70)), red fruit extracts (water (RW), water–ethanol 30:70 (RE30), water–ethanol 70:30 (RE70)) and dark ruby red extracts (water (DRW), water–ethanol 30:70 (DRE30), water–ethanol 70:30 (DRE70)). Data are means ± SD from three independent experiments in which each sample was tested in duplicate. **** *p* < 0.0001.

In vitro tests were carried out on three varieties of *C. mas* L. fruit extracts (water and water–ethanol) at three different dilutions. As shown in Figure 6, the highest proliferation was observed for the YE30 obtaining up to 141.47 ± 6.62% of cell viability for the 1% dilution. Cell viability values were calculated as % of control (cells untreated with extracts). For the NR test (Figure 7) the most favorable cytoprotective properties were observed for the RW *C. mas* L extract obtaining the values of 180.20 ± 8.26% and 189.84 ± 5.98% viability for 5 and 10% dilutions, respectively. In the case of HaCaT cells (Figure 8 and Figure 9), an increase in extract concentration corresponded to an increase in cell viability. In the AB test, the water extracts of the three types of fruits were distinguished by higher proliferation compared to the water–ethanol extracts. The proliferation values were obtained 161.90 ± 7.24%, 155.74 ± 4.96% and 137.98 ± 4.08% for YW, RW and DRW at 10% dilution, respectively. For the NR test (Figure 10), the most favorable cytoprotective properties were observed for DRE30 obtaining 163.66 ± 13.99 and 177.37 ± 6.43% of cell viability at 5 and 10% dilutions, respectively. Moreover, *C. mas* L. fruit extracts at all tested concentrations did not show cytotoxicity. The proliferation values correspond to the values of active compounds, including polyphenols, determined and shown in Table 1, Table 2 and Table 3.

Numerous studies indicate the beneficial effects of phenolic acids and iridoid glycosides found in *C. mas* L. extracts on skin cells. According to Table 7, the highest content of phenolic acids, such as gallic acid, protocatechuic acid, *p*-hydroxybenzoic acid, or *m*-salicylic acid, is found in the red-fruited extract of *C. mas* L. It has been shown that phenolic compounds of plant origin have a positive effect on the functioning of fibroblasts by reducing the production of ROS and increasing the production of collagen. In vitro studies also indicate that these chemicals are able to modify several biomolecular pathways in cells [2,55]. They have been found to mediate protection against oxidants by stimulation of nuclear factors (erythroid-derived 2), factor 2 (Nrf2) and inhibition of mitogen-activated protein kinases (MAPKs); they inhibit the expression of matrix metalloproteinases (MMPs), which are known to interfere with collagen function [56,57]. Gallic and chlorogenic acid are treated as substances that protect against inflammation and aging by reducing the level of ROS, pro-inflammatory cytokines IL-1β and TNF-α [58,59]. Ellagic acid increased the expression level of collagen I and III but decreased the expression levels of MMP-1 and MMP-3 [60]. Moreover, the salicylic acid present in the extracts has keratolytic, comedolytic, and bacteriostatic properties, and studies show no cytotoxicity towards mouse fibroblasts or human keratinocytes [61,62].

These results suggested that phenolic compounds can be useful for supporting fibroblast function, accelerating wound healing, and protecting against UV-induced photoaging. Moreover, studies of the cytoprotective properties of gold nanoparticles synthesized with *C. mas* L. fruit extract showed low toxicity to HaCaT and did not induce additional DNA damage or increased production of inflammatory cytokines [63]. Hyun-Chul et al. showed that the *Cornus walteri* Wangerin leaf extract (CWE) inhibited elastase activity and without cytotoxicity for KeraSkin™-FT reconstructed human skin. Furthermore, CWE significantly reduced MMP-1 expression in UVB-exposed skin [64]. Thus, the tested extracts might be a promising natural ingredient of plant origin in skin care.

### 2.6. Assessment of Anti-Inflammatory Activity

**Figure 11:**
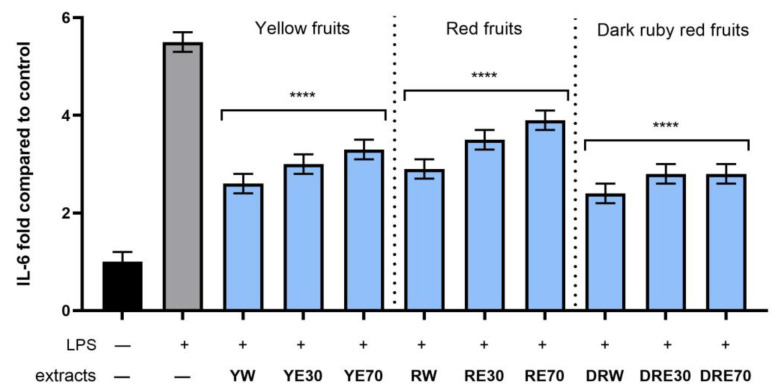
The effect of pretreatment with extracts of *C. mas* L. with yellow, red, and ruby red fruits before exposure to bacterial LPS (10 μg/mL) on the level of interleukin 6 (IL-6) calculated as a percentage in comparison with the untreated control. Analyses were carried out for yellow fruit extracts (water (YW), water–ethanol 30:70 (YE30), water–ethanol 70:30 (YE70)), red fruit extracts (water (RW), water–ethanol 30:70 (RE30), water–ethanol 70:30 (RE70)) and dark ruby red extracts (water (DRW), water–ethanol 30:70 (DRE30), water–ethanol 70:30 (DRE70)). Data are means ± SD from three independent experiments in which each sample was tested in duplicate. **** *p* < 0.0001.

**Figure 12:**
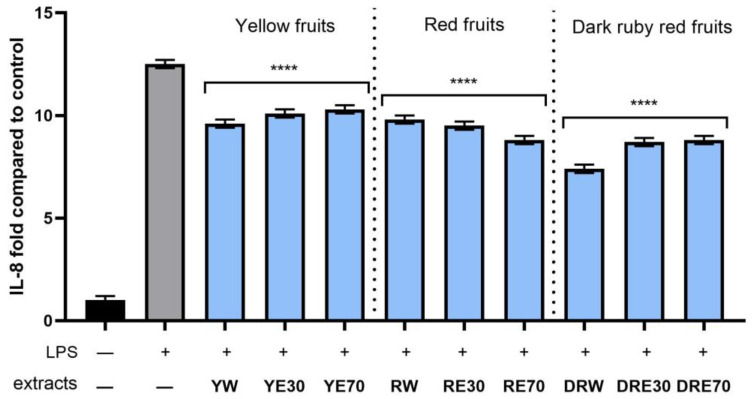
The effect of pretreatment with extracts of *C. mas* L. with yellow, red, and ruby red fruits before exposure to bacterial LPS (10 μg/mL) on the level of interleukin 8 (IL-8) calculated as a percentage in comparison with the untreated control. Analyses were carried out for yellow fruit extracts (water (YW), water–ethanol 30:70 (YE30), water–ethanol 70:30 (YE70)), red fruit extracts (water (RW), water–ethanol 30:70 (RE30), water–ethanol 70:30 (RE70)) and dark ruby red extracts (water (DRW), water–ethanol 30:70 (DRE30), water–ethanol 70:30 (DRE70)). Data are means ± SD from three independent experiments in which each sample was tested in duplicate. **** *p* < 0.0001.

**Figure 13:**
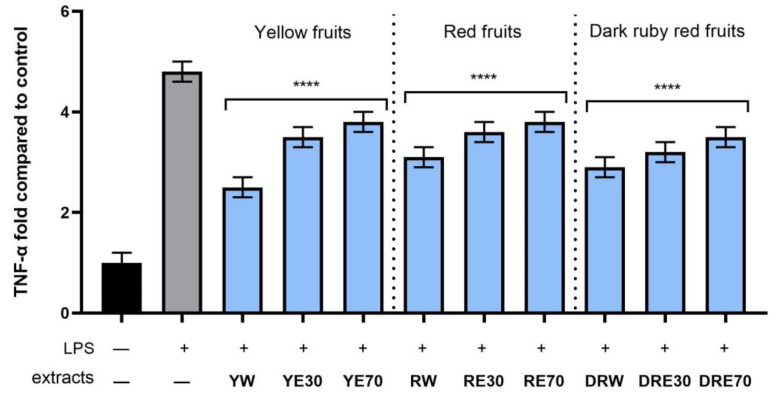
The effect of pretreatment with extracts of *C. mas* L. with yellow, red, and ruby red fruits before exposure to bacterial LPS (10 μg/mL) on the level of tumor necrosis factor α (TNF-α) calculated as a percentage in comparison with the untreated control. Analyses were carried out for yellow fruit extracts (water (YW), water–ethanol 30:70 (YE30), water–ethanol 70:30 (YE70)), red fruit extracts (water (RW), water–ethanol 30:70 (RE30), water–ethanol 70:30 (RE70)) and dark ruby red extracts (water (DRW), water–ethanol 30:70 (DRE30), water–ethanol 70:30 (DRE70)). Data are means ± SD from three independent experiments in which each sample was tested in duplicate. **** *p* < 0.0001.

In order to evaluate the anti-inflammatory effect of all nine *C. mas* L. fruit extracts, the levels of pro-inflammatory interleukins IL-6, IL-8, tumor necrosis factor (TNF-α), and cyclooxygenase-2 (COX-2) were monitored in fibroblast cells treated with the bacterial LPS. For this purpose, fibroblasts (HDF) were pre-treated with tested extracts at a concentration of 10% and exposed to bacterial lipopolysaccharide (LPS). The obtained results showed that LPS is a strong inducer of cytokine production in HDF cells, and the level of interleukins after LPS induction increases significantly (from approximately 4.8 to 12.5-fold) (Figure 10, Figure 11, Figure 12 and Figure 13). The results also indicated that pretreatment of cells with dogwood fruit extracts led to reduced production of all pro-inflammatory factors tested in this study. For all three *C. mas* L. varieties, the strongest inhibition of IL-6 and TNF-α was observed for water extracts. However, in the case of IL-8 and the COX-2 enzyme, it is difficult to clearly indicate which extractant contributes to obtaining extracts with the strongest anti-inflammatory effect, because this effect was different for individual dogwood varieties.

In another study, we also demonstrated the anti-inflammatory properties of the water–glycerin extract from *C. mas* L. (Bolestraszycki cultivar). This study indicated that dogwood extract can significantly reduce the level of pro-inflammatory interleukins (IL-6, IL-8, and TNF-α) in BJ fibroblasts after their exposure to hydrogen peroxide [12,29]. Moldovan et al. also demonstrated that extracts from *C. mas* L. can significantly inhibit the production of pro-inflammatory interleukins, IL-1β and IL-13, in paw tissue, and increase the production of anti-inflammatory interleukin IL-10. Additionally, in vivo examination of paw and liver samples showed that dogwood extract in low doses can alleviate the inflammatory reaction, while in higher doses it can inhibit the exudation of inflammatory cells to the site of inflammation [17].

The anti-inflammatory properties of dogwood fruits are certainly related to the presence of numerous biologically active compounds in these extracts, such as polyphenols, flavonoids, iridoids, and anthocyanins. These compounds have anti-inflammatory properties documented in many studies [17,65,66,67]. The anti-inflammatory properties of dogwood were also confirmed by Choi et al., who examined the activity of cornuside isolated from the fruits of this plant. A study conducted on RAW 264.7 macrophage cells stimulated with LPS showed that this compound inhibits the LPS-induced production of nitric oxide, prostaglandin E2, TNF-α, IL-6, and IL-1beta. Additionally, this compound may reduce the expression of mRNA and proteins of inducible nitric oxide synthase (iNOS) and COX-2. Reduction in iNOS and COX-2 levels is associated with inhibition of NF-κB and negative regulation of extracellular-signal-related kinase (ERK1/2), c-Jun N-terminal kinase (JNK1/2), and p38 phosphorylation [68]. Czerwińska et al. indicated that dogwood extracts may affect the levels of IL-8, IL-1β, and TNF-α in human neutrophils and intestinal epithelial cells Caco-2 [69].

The fact that *C. mas* L. extracts can influence the production of the previously mentioned pro-inflammatory or anti-inflammatory proteins indicates that this plant may be a cosmetics ingredient that inhibits or limits inflammatory processes.

## 3. Materials and Methods

### 3.1. Plant Material and Extraction Procedure

The analyses were carried out on the fruits of three different varieties of *C. mas* L.: Jantarnyj (yellow fruits), Korrałowyj Marka (red fruits) and Bolestraszycki (dark ruby red fruits), which were obtained from a local producer. Three types of extracts were made for each variety: water and water–EtOH in different proportions. Extraction media and process parameters were selected based on experience and information contained in the work of Abubakar and Haque [70]. To prepare water extract, 5 g of fruit and 100 mL of distilled water were used. To prepare water–EtOH extracts in a 70:30 ratio, 5 g of fruit, 70 mL of distilled water and 30 mL of ethanol were used. To obtain water–EtOH extracts in a 30:70 ratio, 5 g of fruit, 30 mL of distilled water and 30 mL of ethanol were used. Extraction was performed using a magnetic stirrer (Chemland, Stargard, Poland) for 14 h. Then, the extracts were sonicated in an ultrasonic bath (Digital Ultrasonic Cleaner) for 30 min at room temperature. The extracts were filtered using Whatman No. 10 filter paper (Thermo Fisher Scientific, Waltham, MA, USA) and stored at 4 °C. Excess prepared extracts were stored at −80 °C.

### 3.2. HPLC Analysis

The analysis of the phenolic acids content was performed using HPLC-UV (Knauer, Germany) based on the procedure described by Al-Rimawi and Odeh with minor modification [71]. The separation was performed on a C18 column (Eurospher II, particle size 5 μM, 125 × 4 mm, Knauer, Berlin, Germany) at a wavelength of 280 nm. The composition of the mobile phase included 1% acetic acid and MeOH (93:7), and the flow rate was 1 mL/min. A volume of 20 µL of sample was injected into the column. The identification of individual compounds was based on the comparison of the retention times of standard substances. The reference substances were purchased from Sigma Aldrich (Steinheim am Albuch, Germany)—caffeic acid, chlorogenic acid, gentisic acid, hypogallic acid, and 3,4-dihydroxybenzoic acid as well as from Merck (Darmstadt, Germany)—3-hydroxybenzoic acid, 4-hydroxybenzoic acid, and gallic acid. The calibration curves were made by preparing solutions of 0.025; 0.0125; 0.00625; 0.00312; 0.00156 and 0.00078%. Next, a calibration curve was plotted for each compound (Table 7). Each sample was analyzed in triplicate. The results presented are the arithmetic mean ± standard deviation (SD).

### 3.3. Antioxidant Activity (DPPH Method) and Total Polyphenol Content (Folin–Ciocalteu Method)

The scavenging activity of DPPH stable free radicals in analyzed extracts as well as acceptor fluids was assessed as previously described [72]. Briefly, 150 μL of the tested samples was mixed with 2850 μL of 0.3 mM DPPH solution (in 96% (*v*/*v*) ethanol). The DPPH working solution was diluted with 70% (*v*/*v*) ethanol until its absorbance at 517 nm was 1.00 ± 0.02. The incubation time of the samples in the dark was 10 min. Analyses at a wavelength of 517 nm were performed with a UV–Vis Spectrophotometer (Hitachi U-5100, Tokyo, Japan). Antioxidant activity determined by the DPPH method was expressed as % of DPPH radical scavenging, which was calculated from the formula:

(1) $$\%DPPHscavenging=\frac{Ac-As}{Ac}\times 100$$

where

As—absorbance of the tested sample;
Ac—absorbance of the control sample.
Each sample was tested in three independent replicates.

The total polyphenol content (TPC) in analyzed extracts as well as acceptor fluids was determined using Folin–Ciocalteu method, according to the methodology described earlier [11]. Briefly, 150 μL of the tested sample, 150 μL of Folin–Ciocalteu reagent (diluted tenfold with water.), 1350 μL of 0.01 M sodium carbonate solution, and 1350 μL of distilled water were mixed and subjected to a 15 min incubation at room temperature. Absorbance measurements were made at a wavelength of 765 nm (UV–Vis Spectrophotometer Hitachi U-5100, Hitachi, Tokyo, Japan). The results were expressed as gallic acid (GA) equivalents in µg·mL^−1^. Three independent measurements were made.

### 3.4. Penetration Skin Studies

The penetration studies were performed using Franz diffusion cells (Phoenix DB-6, ABL&E-JASCO, Vienna, Austria). The donor chamber had a volume of 1 mL, while the acceptor chamber had a capacity of 10 mL and was filled with PBS solution (pH 7.4). During the process, a constant temperature (37.0 ± 0.5 °C) and stirring speed with magnetic stirrers (350 rpm) were maintained in all chambers. Pig skin was used in the experiment because it has similar properties and structure to human skin [73,74]. The pigskin was purchased from a local butcher. Appropriately prepared skin samples, wrapped in aluminum foil, were stored at −20 °C until the experiment was carried out, but no longer than three months. This method of storage allows the skin to maintain its barrier properties [75]. On the day of the experiment, the prepared skin samples were slowly thawed at room temperature (30 min) and then hydrated with PBS buffer (pH 7.4) [76,77]. Skin integrity was checked by impedance testing. This measurement was performed with an LCR 4080 m (Voltcraft LCR 4080, Conrad Electronic, Hirschau, Germany) which operated in parallel mode at an alternating frequency of 120 Hz (error at kΩ < 0.5%). The tips of measuring probes were immersed in the donor and acceptor chamber and filled with PBS buffer (pH 7.4) as described previously [6,78]. Only skin samples with impedance > 3 kΩ were used in the study, as these values correspond to the electrical resistance of human skin [79]. Checked, undamaged pieces of skin were placed in a Franz diffusion chamber between both chambers (donor and acceptor). After the buffer temperature in the acceptor chamber stabilized at 37 °C, 1 mL of the tested plant extract was applied to the donor chamber. To prevent evaporation of the extract during the study, the donor chambers were closed with plastic stoppers. The experiment was conducted over 24 h. After completing the experiment, samples of the acceptor fluid were subjected to HPLC analysis for the content of selected phenolic acids. Based on the obtained concentrations, the cumulative mass (µg) of each phenolic acid was calculated.

The accumulation of phenolic acids in the skin after the 24 h penetration study was determined using a previously described technique [80]. A skin sample from each chamber was thoroughly rinsed in PBS (pH 7.4), and then the skin was sectioned around the diffusion area (1 cm^−2^). Room temperature-dried skin samples were cut into small pieces, and placed in 2 mL of methanol, and then extracted at 4 °C for 24 h. After the extraction was completed, the skin samples were homogenized for 3 min using a homogenizer (IKA^®^ T18 digital ULTRA TURRAX, IKA-Werke GmbH & Co. KG, Staufen Germany), and then the homogenate was centrifuged for 5 min at 3500 rpm [78]. The accumulation of phenolic acids in the skin after the penetration study was calculated by dividing the number of substances remaining in the skin by the mass of the skin sample and expressed as the mass of phenolic acid contained in 1 g of skin (µg·g^−1^).

### 3.5. Detection of Intracellular Levels of Reactive Oxygen Species (ROS)

To assay the capacity of *C. mas* L. fruit extract to generate intracellular level of reactive oxygen species in keratinocytes (HaCaT) and fibroblasts (HDF) cells, the method of Grauzdytė et al. [81] with some modifications, was used. In this method, the fluorogenic dye 2′,7′-dichlorodihydrofluorescein diacetate (H_2_DCFDA; (Sigma Aldrich, Sant Louis, MO, USA) was used. Cells were seeded in 96-well plates at a density of 1 × 10^4^ cells per well and initially cultured before the experiment for 24 h. After this time, the DMEM medium was removed and cells (HaCaT and HDF) were treated with *C. mas* L. extracts dissolved in DMEM at concentrations of 1, 5, 10% for another 24 h. Then, DMEM medium with extracts was changed on 10 µM H_2_DCFDA in serum-free DMEM medium in each well. Then, 5 mM hydrogen peroxide (H_2_O_2_; in a final concentration of 500 µM) dissolved in DMEM without serum was immediately added to the tested samples and the positive sample. After 60 min of incubation, the measurement was taken at an excitation wavelength of λ = 485 nm and an emission wavelength of λ = 530 nm using a microplate reader (FilterMax F5, Thermo Fisher Scientific, Waltham, MA, USA). This analysis included three independent experiments, with each sample being tested in triplicate. 

### 3.6. Cytotoxicity Analysis

#### 3.6.1. Cell Culture

Two types of normal human skin cells, keratinocytes (HaCaT, from CLS Cell Lines Service in Eppelheim, Eppelheim, Germany) and fibroblasts (HDF from CLS Cell Lines Service in Eppelheim, Eppelheim, Germany), were used to evaluate cytotoxic effects. Dulbecco’s Modified Eagle Medium (DMEM from VWR International, Radnor, PA, USA), 10% fetal bovine serum (FBS) from VWR International, Radnor, PA, USA, and 1% antibiotics (Capricorn Scientific, Ebsdorfergrund, Germany) was used. After reaching sufficient confluence, HaCaT and HDF cells were seeded into 96-well plates and cultured for 24 h. The medium was then replaced with *C. mas* extracts at dilutions of 1%, 5%, and 10%, followed by another 24 h incubation period. Cytotoxicity assays were performed on the plates containing the test compounds.

#### 3.6.2. Alamar Blue Assay

The viability of skin cells was initially assessed using the Alamar Blue (AB) method, following the procedure outlined by Page et al. with modifications [82,83]. Following incubation of cells treated with the extracts, the analyzed samples were exposed to a resazurin solution (60 µM). These plates were then incubated at 37 °C for 2 h, after which fluorescence was measured at 570 nm using a FilterMax F5 microplate reader from Thermo Fisher Scientific (Waltham, MA, USA).

#### 3.6.3. Neutral Red Assay

The viability of skin cells was assessed using the Neutral Red Uptake (NR) method, following the procedure described by Borrenfreund et al. with modifications [83]. After the incubation period, the analyzed samples were extracted from the wells and exposed to Neutral Red dye (40 µg/mL). The plates were then incubated for 2 h at 37 °C. Following incubation, the cells were washed with phosphate-buffered saline before adding 150 µL of decolorizing buffer (50% ethanol, 1% acetic acid, 49% water). Absorbance measurements were taken at a wavelength of 540 nm using a FilterMax F5 microplate reader from Thermo Fisher Scientific (Waltham, MA, USA).

### 3.7. Assessment of Anti-Inflammatory Activity

In order to assess the anti-inflammatory properties of *C. mas* L. extracts, the levels of IL-6, IL-8, TNF-α and COX-2 were assessed in fibroblasts (HDF) exposed to bacterial lipopolysaccharide (LPS, 10 µg/mL) from *Escherichia coli* O111:B4 for 24 h. The cells were treated simultaneously with all nine different dogwood extracts at a concentration of 10%. Commercially available enzyme-linked immunosorbent assays (Elabscience Biotechnology Inc., Houston, TX, USA) were used in the analysis. The analysis was performed according to the instructions provided by the manufacturer and the absorbance was measured using a microplate reader (FilterMax F5, Thermo Fisher Scientific, Waltham, MA, USA) at λ = 450 nm. Control cells were fibroblasts not treated with LPS or extracts, and positive controls were HDF cells treated with LPS (without the addition of extracts).

### 3.8. Statistical Analysis

Results are presented as the mean ± standard deviation (SD). Each value is the average of three replicates. A one-way analysis of variance (ANOVA) was used. The significance of differences between individual *C. mas* L. extracts was assessed using the Tukey test (α < 0.05), while differences in the effects of individual extract concentrations compared to the control were assessed using Dunnett’s post-test (α < 0.05). Statistical calculations were conducted using Statistica 13 PL software (StatSoft, Kraków, Poland) and GraphPad Prism 8.4.3 (GraphPad Software, Inc., San Diego, CA, USA).

## 4. Conclusions

The research conducted as part of this study allowed us to compare the antioxidant, anti-inflammatory, and cytotoxic effects of three varieties of *C. mas* L. with different fruit colors. The results demonstrated differences in the biological activity of the tested extracts, indicating the strongest effect of dark ruby red-colored fruits compared to red and yellow fruits. The obtained results also showed a positive effect of the tested extracts on the proliferation and metabolic activity of skin cells in vitro. The anti-inflammatory properties of dogwood were also confirmed by measuring the level of pro-inflammatory cytokines in LPS-treated fibroblasts. The study also showed that the type of extractant used in the extraction process has a significant impact on both the amount of extracted phytochemicals and the biological activity of the extracts. It has been shown that the use of a mixture of water and ethanol increases the amount of extracted phytochemicals compared to the use of water as an extractant. Moreover, analyses using pig skin indicated differences in skin penetration by individual phenolic compounds present in *C. mas* extracts. It should be noted, however, that the effect of cosmetics containing dogwood extracts may be slightly different than the effect of the extracts themselves. This is a result of the presence of many lipophilic and hydrophilic compounds in cosmetic formulations, which may influence the final effect of the cosmetic preparation. Therefore, further research is required on the properties of cosmetics with dogwood extracts to confirm the possibility of multidirectional action of dogwood in this type of preparations.

## Figures and Tables

**Table 1:** Values of phenolic acids in dark ruby red-fruited *C. mas* L. variety. Mean (±standard deviation), (*n* = 3); different letters indicate significant differences between t individual extracts within each *C. mas* L. variety; α = 0.05.

Phenolic Acid	DRW	DRE30	DRE70
	µg/mL		
gallic acid	32.96 ± 1.59 a	42.07 ± 2.76 a	38.72 ± 1.39 a
protocatechuic acid	15.29 ± 1.64 a	18.36 ± 1.24 a	17.06 ± 1.10 a
gentisic acid	4.82 ± 0.24 a	6.45 ± 0.27 a	8.95 ± 0.18 b
*p*-hydroxybenzoic acid	10.42 ± 1.77 a	15.75 ± 5.88 b	17.66 ± 0.97 b
hypogallic acid	1.85 ± 0.37 b	2.36 ± 0.14 b	0.20 ± 0.12 a
*m*-salicylic acid	17.65 ± 0.87 c	4.28 ± 1.67 a	12.79 ± 0.92 b
caffeic acid	9.64 ± 0.22 b	3.79 ± 0.08 a	14.43 ± 0.97 c
chlorogenic acid	1.73 ± 0.06 a	4.00 ± 0.41 a	26.94 ± 1.87 b

**Table 2:** Values of phenolic acids in red-fruited *C. mas* L. variety. Mean (±standard deviation), (*n* = 3); different letters indicate significant differences between individual extracts within each *C. mas* L. variety; α = 0.05.

Phenolic Acid	RW	RE30	RE70
	µg/mL		
gallic acid	21.26 ± 1.24 a	28.91 ± 0.96 b	33.82 ± 2.02 c
protocatechuic acid	5.71 ± 0.24 a	13.30 ± 1.26 c	8.37 ± 0.15 b
gentisic acid	n.d.	n.d.	n.d.
*p*-hydroxybenzoic acid	8.93 ± 0.79 a	9.75 ± 0.23 a	24.51 ± 3.10 b
hypogallic acid	0.92 ± 0.23 a	n.d.	n.d.
*m*-salicylic acid	13.61 ± 1.36 a	23.59 ± 0.70 b	14.99 ± 0.25 a
caffeic acid	8.42 ± 0.49 a	9.27 ± 0.30 a	8.78 ± 0.58 a
chlorogenic acid	8.23 ± 1.22 a	10.97 ± 0.73 b	n.d.

n.d.—not detected.

**Table 3:** Values of phenolic acids in yellow-fruited *C. mas* L. variety. Mean (±standard deviation), (*n* = 3); different letters indicate significant differences between individual extracts within each *C. mas* L. variety; α = 0.05.

Phenolic Acid	YW	YE30	YE70
	µg/mL		
gallic acid	28.46 ± 1.26 b	35.58 ± 1.55 c	16.83 ± 0.35 a
protocatechuic acid	18.48 ± 1.35 b	33.01 ± 1.35 c	12.19 ± 0.88 a
gentisic acid	8.23 ± 0.92 a	11.35 ± 1.68 b	5.97 ± 0.35 a
*p*-hydroxybenzoic acid	11.48 ± 1.18 a	11.00 ± 1.38 a	9.53 ± 0.47
hypogallic acid	1.52 ± 0.17 a	2.01 ± 0.41 a	1.85 ± 0.41 a
*m*-salicylic acid	7.58 ± 1.00 a	19.80 ± 3.01 b	n.d.
caffeic acid	n.d.	13.61 ± 0.67 b	3.48 ± 0.04 a
chlorogenic acid	4.36 ± 0.55 a	35.45 ± 2.18 b	4.08 ± 0.53 a

n.d.—not detected.

**Table 4:** The content of phenolic acids in acceptor fluid after 24 h penetration study in dark ruby red-fruited *C. mas* L. variety. Different letters indicate significant differences between individual extracts within each *C. mas* L. variety; (*n* = 3); α = 0.05.

Extracts	Acceptor Fluid after 24 h of Penetration (µg)							
	Gallic Acid	Protocatechuic Acid	Gentisic Acid	*p*-Hydroxybenzoic Acid	Hypogallic Acid	*m*-Salicylic Acid	Caffeic Acid	Chlorogenic Acid
DRW	1.25 ± 0.34 a	3.01 ± 0.11 a	3.97 ± 0.28 b	0.32 ± 0.35 a	n.d.	n.d.	n.d.	n.d.
DRE30	2.36 ± 0.12 c	3.06 ± 0.19 a	2.01 ± 0.46 a	n.d.	n.d.	n.d.	n.d.	n.d.
DRE70	1.81 ± 0.05 b	5.86 ± 0.52 b	3.55 ± 0.70 b	n.d.	n.d.	n.d.	n.d.	n.d.

n.d.—not detected.

**Table 5:** The content of phenolic acids in acceptor fluid after 24 h penetration study in red-fruited *C. mas* L. variety. Different letters indicate significant differences between individual extracts within each *C. mas* L. variety; (*n* = 3); α = 0.05.

Extracts	Acceptor Fluid after 24 h of Penetration (µg)							
	Gallic Acid	Protocatechuic Acid	Gentisic Acid	*p*-Hydroxybenzoic Acid	Hypogallic Acid	*m*-Salicylic Acid	Caffeic Acid	Chlorogenic Acid
DRW	3.36 ± 0.64 a	1.91 ± 0.06	n.d.	7.48 ± 0.48 b	n.d.	n.d.	n.d.	n.d.
DRE30	2.35 ± 0.07 a	0.64 ± 0.10 a	n.d.	7.08 ± 0.93 b	n.d.	n.d.	n.d.	n.d.
DRE70	3.13 ± 0.39 a	0.69 ± 0.11 a	n.d.	4.21 ± 0.81 a	n.d.	n.d.	n.d.	n.d.

n.d.—not detected.

**Table 6:** The content of phenolic acid in acceptor fluid after 24 h penetration study in yellow-fruited *C. mas* L. variety. Different letters indicate significant differences between individual extracts within each *C. mas* L. variety; (*n* = 3); α = 0.05.

Extracts	Acceptor Fluid after 24 h of Penetration (µg)							
	Gallic Acid	Protocatechuic Acid	Gentisic Acid	*p*-Hydroxybenzoic Acid	Hypogallic Acid	*m*-Salicylic Acid	Caffeic Acid	Chlorogenic Acid
DRW	1.30 ± 0.13 a	0.62 ± 0.05 a	1.13 ± 0.12 a	n.d.	n.d.	n.d.	n.d.	n.d.
DRE30	1.79 ± 0.30 a	1.04 ± 0.06 b	2.53 ± 0.26 b	n.d.	n.d.	n.d.	n.d.	n.d.
DRE70	1.20 ± 0.14 a	0.66 ± 0.11 a	1.45 ± 0.05 a	1.30 ± 0.15 a	n.d.	n.d.	n.d.	n.d.

n.d.—not detected.

**Table 7:** Parameters of standards determined in *C. mas* L. extracts.

Standard	Correlation Coefficient (R^2^)	Linear Function Coefficient (y = ax + b)
gallic acid (3,4,5-trihydroxybenzoic acid)	0.9999	y = 157,710x − 4.1682
protocatechuic acid (3,4-dihydroxybenzoic acid)	0.9991	y = 1710.3x + 0.059
gentisic acid (2,5-dihydroxybenzoic acid)	0.9998	y =1436.7x − 0.0256
*p*-hydroxybenzoic acid (4-hydroxybenzoic acid)	1.0000	y = 8527.4x + 0.1935
hypogallic acid (2,3-dihydroxybenzoic acid)	1.0000	y = 51,301x + 1.8318
*m*-salicylic acid (3-hydroxybenzoic acid)	0.9998	y = 7971x + 0.8098
caffeic acid (3,4-dihydroxycinnamic acid)	0.9999	y = 20,803x − 0.6458
chlorogenic acid (3-caffeoylquinic acid)	0.9998	y = 8253.9x + 2454

## Data Availability

The data presented in this study are available on request from the corresponding author.
